# Oleogel-Based Systems for the Delivery of Bioactive Compounds in Foods

**DOI:** 10.3390/gels7030086

**Published:** 2021-07-07

**Authors:** Tiago C. Pinto, Artur J. Martins, Lorenzo Pastrana, Maria C. Pereira, Miguel A. Cerqueira

**Affiliations:** 1 LEPABE—Laboratory for Process Engineering, Environment, Biotechnology and Energy, Faculty of Engineering, University of Porto, Rua Dr Roberto Frias, 4200-465 Porto, Portugal; tiagoccpinto@gmail.com (T.C.P.); mcsp@fe.up.pt (M.C.P.); 2 INL—International Iberian Nanotechnology Laboratory, Avenida Mestre José Veiga, 4715-330 Braga, Portugal; artur.martins@inl.int (A.J.M.); lorenzo.pastrana@inl.int (L.P.)

**Keywords:** oleogelation, emulsification, bioactive compounds, delivery, bioavailability

## Abstract

Oleogels are semi-solid materials containing a large fraction of liquid oil entrapped in a network of structuring molecules. In the food industry, these formulations can be used to mimic fats and to deliver bioactive compounds. In the last decade, there has been increasing interest in these structures, not only from a scientific point of view, i.e., studying new molecules, methodologies for gelification, and new structures, but also from a technological point of view, with researchers and companies exploring these structures as a way to overcome certain challenges and/or create new and innovative products. One of the exciting applications of oleogels is the delivery of functional molecules, where the incorporation of oil-soluble functional compounds can be explored not only at the macroscale but also at micro- and nanoscales, resulting in different release behaviors and also different applications. This review presents and discusses the most recent works on the development, production, characterization, and applications of oleogels and other oleogel-based systems to deliver functional molecules in foods.

## 1. Introduction

Recently, food systems have undergone significant changes due to advances in food processing, and the current fast-paced way of living has increased the demand for more available and affordable food products. Traditional diets, featuring whole or minimally processed foods, were gradually replaced in modern society by industrialized and pre-prepared food products [1]. For technological reasons, saturated fats are common in many processed foods. The addition of fats to processed food products allows for the exhibit of interesting organoleptic properties, such as texture, mouth-feel, and flavor. This creates a barrier to its substitution in foods since it can significantly overcome the mentioned characteristics and, consequently, hinder the overall eating pleasure. The intake of essential fatty acids and antioxidants naturally occurring in traditional diets has consequently given place to the consumption of unhealthy fats, which are deeply related to health disorders, such as coronary heart disease, inflammation, oxidative stress, and metabolic syndrome [2]. Currently, food trends have been shifting towards healthy eating and plant-based diets. Consumers are increasingly aware of the negative environmental impacts caused by animal source food production and of the dangers that are associated with the consumption of overly processed animal products, therefore generating new challenges and roadblocks in the food sector in order to meet consumers’ demands [3].

The pursuit of healthy substitutes for fats is not recent; the hydrogenation process has been developed as early as the beginning of the 20th century, as a strategy for replacing the consumption of animal saturated fats and cholesterol intake by vegetable and marine oils, rich in healthy polyunsaturated fatty acids (PUFAs). This process changes the degree of saturation and confers to these oils the firmness and plasticity desired by food manufacturers and consumers [4]. During the second part of the century, it took over the everyday diet in the United States and several other western countries. At first, this seemed to be a positive alternative to saturated fats, thus being promoted by health advocates. However, a side effect of the incomplete hydrogenation of fats is the isomerization of the remaining double bonds, converting them to the trans-configuration [5]. In the 1990s, the first studies on the dietary impact of trans fats appeared, associating them with unfavorable effects on the serum lipoprotein profile. Trans fats were proven to not only raise low-density lipoprotein (LDL) cholesterol levels but also to lower high-density lipoprotein (HDL) cholesterol levels [5]. Since then, the World Health Organization (WHO) has strived to eliminate artificial trans fats from food supply chains, with some countries being pioneers in entirely eradicating the manufacture of food products using trans fats [6]. In the United States, trans fats are no longer considered Generally Recognized as Safe (GRAS), and the Food and Drug Administration (FDA) and the European Union (EU) have established the deadline of 1 January 2021 for food producers to adopt the new guidelines regarding trans fats [7,8].

The liquid nature of vegetable oils limits their utility in some applications, since they do not share the same properties as solid fats [9]. Considering this, and within governments’ frameworks for removing unhealthy fats from the market, new developments in oil structuring systems have appeared in the food industry, with oleogels being at the forefront of the scientific quest. Oil structuring is based on the formation of a gelator network that allows for the formation of a self-standing thermo-reversible viscoelastic structure without affecting the chemical structure of the oil. Unlike the hydrogenation process, oleogels are a viable way of structuring oils that are rich in PUFAs without undermining their health potential [2]. The acceptability of oleogels by consumers depends on their capability of mimicking the characteristics of solid fats. Likewise, oleogels can be tailored to match a specific purpose, with structural and textural characteristics being an essential factor. Other than the replacement of non-healthy fats with healthy oils, oleogels can also have added nutritional value through the addition of bioactive compounds to the formulation. This functionality can be an advantage in nutritional terms and in making the product more attractive in terms of stability and shelf-life.

The main hindrance to using oleogels in food matrices is their low compatibility with water-based food products. As a way of circumventing this problem, oleogels can be used in emulsion templates by applying conventional emulsification techniques, such as high-shear homogenization [10]. The interplay between oleogelation and emulsification expands the possible applications of oleogels in the food industry, making them a suitable tool for many new products. These biphasic systems are capable of constituting a structured system with the benefit of decreasing the fat content of a product and might be more suitable for certain applications as opposed to pure oleogels [11]. Furthermore, this approach may broaden their potential to introduce hydrophilic bioactive compounds, rather than lipophilic compounds, or even allowing for the co-encapsulation of bioactive compounds in both phases of an oleogel-based emulsified system.

## 2. Hydrogels, Oleogels, Bigels, and Emulgels

**Figure 1:**
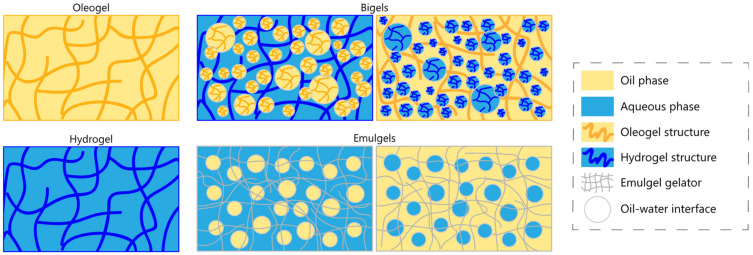
Schematic representation of oleogels, hydrogels, bigels, and emulgels structures.

Gels represent a type of colloid that consists of a solid-like three-dimensional network, in which a liquid phase is entrapped. A gel can be defined as a coherent system of at least two components, which exhibits mechanical properties of a solid, where both the dispersed component and the dispersion medium extend themselves continuously throughout the whole system [12]. Hermans proposed this definition as the author of the first recorded effort to connect the macroscopic and microscopic properties of a gel, which helped to define its hybrid characteristics between liquid and solid materials [12]. Gel formulations can be divided into two major classes according to the solvent used for their production; hydrogels refer to the case where the liquid phase is water, and organogels (or oleogels) when the dispersed liquid is an organic solvent and is structured by an organogelator [13]. Hydrogels consist mainly of a hydrophilic polymeric network, which can absorb high quantities of liquid (Figure 1). The capability of hydrogels of absorbing fluids arises from the hydrophilic functional groups on the backbone of the crosslinked polymer chains, therefore facilitating the diffusion of liquid and important solute molecules [14]. Hydrogels have certain characteristics, i.e., their hydrophilicity, flexibility, elasticity, softness, and high swelling capability, which allow them to be applied in a plethora of different applications [15]. Usually, hydrogels are commonly associated with pharmaceutical applications since their features make them highly efficient for transdermal drug delivery [13]. As such, many novel hydrogel-based delivery matrices have been designed for pharmaceutical and medical fields, playing a vital role in diagnosis and treatment [16,17,18]. Recently, hydrogels have been explored in other areas, such as tissue engineering, cosmetics, and food technology, with an increasing number of publications on the subject [19,20,21,22,23,24].

Organogels are semi-rigid formulations considered bicontinuous systems, comprising two phases: the gelator and the organic solvent (Figure 1). The gelator, when used in the formulation of organogels in concentrations of <15%, may experience physical and chemical transformations that create self-assembled structures; these structures entangle with each other, forming a three-dimensional network. The organic solvent is retained and immobilized within the spaces of the gelator network. If the used solvent is a liquid oil, then the term oleogel is also appropriate for these formulations. Therefore, oleogels allow properties to be explored that hydrogels are not compatible with, such as hydrophobicity of compounds [13]. One of the main advantages of oleogels is the possibility of carrying lipophilic bioactive compounds, which is of great utility in both pharmaceutical and food applications [11]. The combined action between structure and health benefits supports the important role that oleogels can have in novel food products, as they can be tailored to meet the ideal properties for a food product, acting as a healthy substitute for solid fats [25,26]. Great attention from the scientific and industrial communities towards oleogels has risen since they were first suggested as a possible substitute for fats.

Certain types of gels are developed in a way that combines certain characteristics of both hydrogels and oleogels. These hybrid gels, or bigels, are systems that, in general, contain two immiscible liquid phases that are individually stabilized by independent gelators (Figure 1) [13]. Bigels display the merits of both the aqueous and oil phase, including the ability to deliver simultaneously hydrophilic and lipophilic active agents, and improved viscoelasticity [26]. Bigels have been mainly applied in cosmetic/pharmaceutical formulations, since their hybrid characteristics are proven to maximize moisturizing benefits for the skin and simplify the penetration of active agents to deeper layers of the skin tissue [27,28,29,30]. These benefits may also be very valuable for food applications in the delivery of bioactive compounds [31]. Despite their recognized potential, bigels are still underexplored in terms of their microstructure, which has been scrutinized only in recent years [32].

On the other hand, emulgels may be considered a type of hybrid between emulsions and gels (Figure 1). Emulgels result from an initial emulsification process followed by a gelation process, through gelation and/or crosslinking of the compounds that are present in the mixture. The amphiphilic behavior of the emulgels, which is potentiated by the hydrophilic and lipophilic affinities of its constituents, makes them a good option for the delivery of active agents, in a similar way to bigels [26,33,34,35]. In this way, emulgels have been at the forefront of the topical and transdermal delivery of drugs, due to characteristics such as their easy removability, emollient action, ease of extrusion, and spreadability. However, these are the same characteristics that hinder emulgels from being as appealing for food applications, in addition to their stickiness and proneness to phase separation [13,14]. Figure 1 presents a schematic representation of oleogels, hydrogels, bigels, and emulgels structures.

## 3. Oleogel Preparation for Food Applications

Semi-solid fat products, such as ice creams, chocolates, butter, margarine, or other spreads, owe many of their structural properties to saturated and trans fatty acids. These fatty acids, present in the form of triacylglycerols (TAGs), form a three-dimensional colloidal fat crystal network. Upon crystallization, the TAGs aggregate to form fat crystals, and these crystals aggregate into flocs. This resulting fat crystal network entrains the liquid components of the fat, preventing their exudation [36]. However, the growing proof that the intake of saturated and trans unsaturated fats is associated with cardiovascular disease, type II diabetes, high cholesterol, and ischemic stroke risk has led to legislative reformulation regarding these types of fats, compelling food producers to come up with alternative ingredients [37,38]. The issue with replacing them is that it is very difficult to do so without compromising the overall properties of the above-mentioned products.

**Figure 2:**
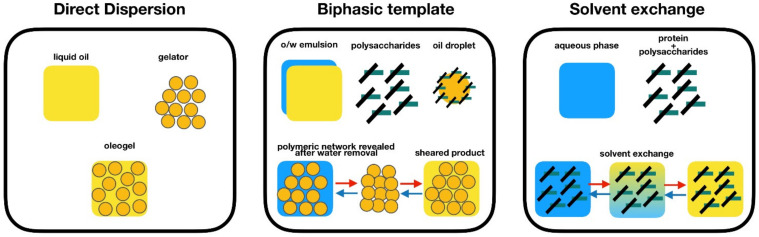
Strategies for the formation of oleogels. Reprinted by permission from Springer: [39].

The process of oleogelation aims at conferring to a liquid oil the distinctive features of solid fat without needing a large amount of saturated and trans-fat to achieve it. Therefore, oleogelation aims to create an alternative structuring process, envisioning an edible oil structure that mimics the fat crystal network. Several gelation methodologies have been studied, generating oleogels with very interesting properties [39] that can be divided into direct dispersion, biphasic template, and solvent exchange methods (Figure 2).

Substances that gel edible oils can be roughly divided into two categories based on their molecular weight: low molecular-mass organic gelators (LMOGs), and polymeric gelators [36]. Most of the early work on oleogels focused on LMOGs, such as waxes, sterol-based gelators, fatty acid derivatives, and monoacylglycerols [39]. The polymeric gelation approach is still underexplored, mainly because most of the food-grade polymers at our disposal are hydrophilic, and very few can be used to produce oleogels. The types of gelators that are being used have different gelation mechanisms, which result in certain structural properties and macroscopic features. The used oil phase also plays a relevant role since the fatty acid profile can influence the oleogels’ final rheological, textural, and visual properties [2]. Based on this, the selection of components and the gelation strategy for the production of the oleogel should be made envisioning the properties desired for the said product.

### 3.1. Direct Dispersion

Direct dispersion methodologies consist of a direct dispersion of the oleogelator into the liquid oil at temperatures above the melting point. This is followed by a cooling period, during which the gelator network is formed, entrapping the oil in a solid structure, thus forming the oleogel. Using a direct approach, the gelation mechanism itself can produce two different types of networks, depending on the type of structurant used: crystallite conformations, or self-assembled networks [2].

#### 3.1.1. Crystallite Conformations

Different types of gelators can originate crystallite conformations. Lipid-based gelators, e.g., waxes, monoacylglycerols (MAGs), fatty acids, and fatty alcohols, are commonly used via a direct dispersion strategy and form a crystalline network. This is a very commonly used process for oleogel synthesis, since it is very similar to the traditional process of oil structuring using solid fats. Steps of nucleation, crystal growth, aggregation, and network formation are involved in both processes [11]. However, conventional lipid structuring is based on creating a TAGs hardstock that, in order to achieve efficient oil structuring, is required to be added in a fraction of about 20% of the oleogel [40]. The use of gelators such as waxes, wax esters, and MAGs is a very interesting and cost-effective alternative, due to the lower critical concentration needed to induce oleogelation. Typically, these compounds only need a minor purification or concentration step to convert them into functional lipid gelators [40]. Moreover, the wax composition greatly influences the molecular organization of the oleogel structure, making them a viable option for many oleogels with very different thermal and rheological properties. In particular, alkyl ester chain length and degree of saturation influence crystal development, causing changes at the crystalline network level. Other factors such as the presence of impurities that affect wax crystal morphology, and shear and cooling rate that have shown to effectively modify the crystal morphology of the wax, can be used to tailor the gel’s properties to certain applications [41]. The conformational arrangement and crystal type also depend on the chemical composition of the gelator; in that regard, waxes, MAGs, and other fatty acid derivatives can form oleogels with different crystal morphologies, even though the preparation is similar [42,43].

#### 3.1.2. Self-Assembled Networks

Self-assembled networks can be originated by essentially four kinds of gelators: LMOGs, polymers, colloidal silicon dioxide particles, and lecithin [11,44]. Regarding LMOGs, sterol-based oleogels are among the most interesting and widely studied ones, due to sterols’ capability to produce highly stable oleogels and their health-related benefits. One example is the combination of oryzanol and phytosterol, which results in a tubular-shaped arrangement that can form oleogels with enhanced mechanical properties [45]. These are coined self-assembled fibrillar networks (SAFiNs), as the gelation mechanism consists of the formation of a set of fibrils that present unidirectional growth and entangle with each other, forming a fibrillar network. The final length of the fibers is highly dependent on the environmental conditions, such as cooling rate and storage temperature, so these parameters can be optimized depending on the intended characteristics of the oleogel [25]. Because these systems form thin fibrils, the structural network is translucent even with high concentrations of oleogelator, allowing the oleogel to have a similar color to the oil, which may be an appealing characteristic for certain applications where the visual impact of the product plays a determinant role in customer acceptability [46]. Not only are these compounds approved for use in food applications, but they have been proven to actively have an impact in lowering blood LDL cholesterol and reducing the risk of coronary heart disease, according to the European Food Safety Authority (EFSA) [47]. Other LMOGs such as 12-hydroxystearic acid and ricinoleic acid are examples of oleogelators that can be incorporated either as the only gelator or in mixed systems [44,48,49].

As of now, the only known polymer that can act as an oleogelator through the direct method is ethylcellulose (EC) [25]. After complete solubilization of the EC in the liquid oil at an above glass transition temperature (~140 °C), the polymer softens, and there is partial solubilization in the oil. The cooling period follows, during which the polymer returns to its rigid form, inducing the formation of hydrogen bonds that result in a three-dimensional polymer network. This network retains the liquid oil and, depending on the viscosity grade, confers higher or lower viscoelastic properties to the oleogel. If these requirements are not met, the risk of incomplete gelation is high [39].

Colloidal silicon dioxide (CSD) particles are among the inorganic particles that have relevant applications in food, cosmetic, and pharmaceutical fields [50,51]. They are approved for use as a food additive and were reported to function as a gelator for vegetable oils in oleogel and bigel systems. When added to the liquid oil in at least 10%, the CSD particles result in fractal aggregates that form a network stabilized by hydrogen bonds and electrostatic interactions [52].

Another possibility for the development of self-assembled networks is the use of lecithin. This amphiphilic molecule, when surrounded by a non-polar organic liquid, like an oil, forms reverse spherical micelles. Lecithin-based oleogels require the addition of a polar solvent (e.g., water); this promotes the uniaxial growth of the micelles and turns them into elongated tubular structures that subsequently entangle to form a three-dimensional network. These crosslinked tubules entrap the oil phase and produce a gel, very similar to the polymer oleogels [53]. Moreover, lecithin has been proven to interact with other gelators, such as waxes, phytosterols, and EC, as co-gelator in direct dispersion methods. In some situations, the synergistic relationships between the gelators in these multi-component oleogels exhibited improved rheological properties when compared to the single-component oleogels [54,55,56].

### 3.2. Indirect Dispersion

Indirect approaches to oil structuring can be interesting and have been gaining recognition in recent years. The great advantage of indirect methods is the fact that it widens considerably the types of oleogelators that can be used, with recent publications introducing proteins, polysaccharides, and polymers other than EC as oil gelators. The indirect methods include biphasic emulsion-based strategies and solvent exchange methodologies [57].

#### 3.2.1. Emulsion-Template Methodologies

The emulsion-template approach is a promising method for using hydrophilic gelators that cannot be directly dispersed in oil to achieve the network structure necessary for oleogels. This methodology involves the preparation of an emulsion stabilized by a hydrophilic gelator, followed by the removal of the hydrophilic solvent. The result is a dried structure, where the hydrophilic gelator network acts as a building block for the oil fraction, forming an oleogel [58]. This methodology broadens the range of food-grade polymers that can be used for oleogelation, since most of them are hydrophilic. However, some of them exhibit amphiphilic properties (e.g., proteins) that can be useful in the interaction with the oil phase.

For example, chitin is one of the polymers most present in nature and is highly biocompatible and biodegradable; however, it is reported to be inefficient as a sole gelator component, and its direct dispersion results in poorly stable gels [59]. Adversely, it has been reported that, when used in combination with a surfactant, a stable oleogel can be produced and can acquire very interesting properties. Namely, phosphatidylcholine, enzymatically modified phosphatidylcholine and sorbitan monostearate have been used in combination with chitin, all of them inducing improvements at the level of gel strength to different extents. The temperature sensitivity of the oleogels was also found to be related to the type of surfactant used, playing an important role in the melting behavior of the oleogel [60]. As such, chitin can be a highly versatile oleogelator, as the surfactant can tailor the oleogels for many desired properties and applications.

Cellulose derivatives, besides EC, can also be used in oil structuring applications by the emulsion-based approach. Well-documented food-grade options include hydroxypropyl-methylcellulose (HPMC) and carboxymethylcellulose (CMC). HPMC is usually used as a foaming agent that, once dried, has very interesting oil sorption characteristics and consequently forms solid-like oleogels once sheared [61]. CMC, in combination with regenerated cellulose (RC), was used as a stabilizer for oil-in-water emulsions that, after water removal through freeze-drying, created a structured oleogel system [62].

Another widely explored option is the use of proteins as oleogelators. Proteins are more recognized due to their potential as gelators in hydrogels rather than in oleogels, due to their predominantly hydrophilic characteristics. One of the possibilities for the synthesis of a protein oleogel is starting by preparing an emulsion using proteins as the emulsifying agent and removing the water phase. The properties of the interface can be strengthened with the addition of a polysaccharide, with gelatin and xanthan gum being highly popular. This is usually a required step to prevent the coalescence of the oil droplets and form a stable oleogel [63].

#### 3.2.2. Solvent Exchange Methodologies

The solvent exchange method can also be used to create protein and polysaccharide building blocks for oleogelation. This procedure is based on the formation of a protein network within an aqueous medium (hydrogel). The polarity of the solvent is decreased by the introduction of an intermediate organic solvent (i.e., acetone or tetrahydrofuran). Finally, the organic solvent is substituted by an oil, through a sequence of dipping or immersion steps [39,63]. De Vries et al. [64] developed an approach using whey protein isolate as an oleogelator in sunflower oil, obtaining oleogels that were much stiffer than the hydrogels that served as a starting point. The oil holding capacity was affected by factors such as the properties of the protein network, the polarity of the intermediate solvent, and the kinetics of the solvent exchange methodology, which further confirms the versatility of the method. Similarly, the solvent exchange method was applied to polysaccharide-based hydrogels using κ-carrageenan hydrogels. This procedure featured an intermediate step of immersion in alcohol solutions, followed by supercritical CO_2_ drying to avoid polymeric collapse and obtain stable aerogels. These aerogels were later dipped in sunflower oil to induce the uptake of oil [65]. The κ-carrageenan network proved to be very effective in the uptake of oil and sets a strong precedent for the use of other biopolymers as oleogelators.

This kind of stepwise methodology can come up as an effective way of benefiting from the thermal and mechanical properties of many oleogelators in one single system. Furthermore, it considerably widens the type of gelators that can be used for the preparation of oleogels, which consequently has an impact on the final product. The customization of the oleogels can also be performed through several steps along the process, particularly the intermediate solvents that are used and the kinetics of the process [39].

## 4. Oleogel-Based Emulsion Systems Using Food-Grade Components

During the last 10 years, food-grade oleogels have been the subject of much research interest worldwide, and the variety of gelators that harbor different gel-formation mechanisms have led to numerous publications [2,36,66]. As an effort to expand the scope of oleogel-based food products, the development of oleogel-based systems that comprise a water fraction has started to gain attention. Moreover, the development of hybrid structures can help to modulate the extent of lipolysis and to increase lipid digestibility, while maintaining the solid-like structure that distinguishes the oleogels. Understanding the interplay between emulsification and oleogelation is fundamental [10]. In this section, the focus is on oleogel-derived emulsified systems, where the oil phase is structured, whereas the aqueous phase is not.

### 4.1. Single Emulsions

Emulsions are colloidal dispersions comprising two immiscible liquids, in which one of them is dispersed in a continuous liquid phase of different composition. The continuous phase is referred to as the ‘external phase’, while the dispersed phase can be called the ‘internal phase’. Considering that one of the liquids is aqueous (polar), and the other is non-polar, typically being an oil, two types of emulsions can be distinguished:

- oil-in-water (O/W)—oil droplets are dispersed in the continuous water phase;
- water-in-oil (W/O)—water droplets are dispersed in the continuous oil phase.

**Figure 3:**
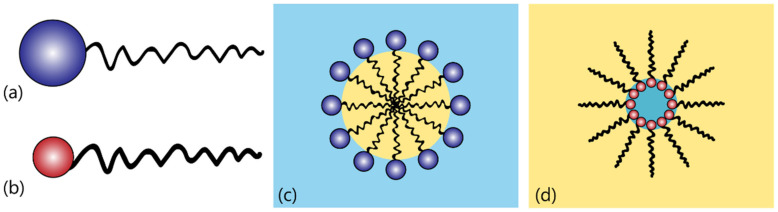
Illustration of the surfactant molecules’ arrangement in single emulsions: (**a**) hydrophilic emulsifier molecule (high HLB); (**b**) lipophilic emulsifier molecule (low HLB); (**c**) micelle in an O/W emulsion; (**d**) reverse micelle in a W/O emulsion.

Essentially, most emulsions comprise the two liquid phases and an emulsifying agent, which stabilizes the emulsion and may form a protective layer on the interface between the two liquids, keeping the droplets from coalescing and preventing the emulsion from breaking. Typically, the emulsifying agents are surfactants, which are a class of compounds that act as surface-active agents. Their amphiphilic behavior provides them with the capability of associating in micelles [67]. In the case of an O/W emulsion, the emulsifier molecules arrange themselves with the non-polar tails extending into the oil and the polar heads facing the water, forming micelles; in the case of a W/O emulsion, the emulsifier’s orientation is reversed, forming reverse micelles [68]. These two situations are illustrated in Figure 3.

Several empirical approaches predict the surfactant positioning at the interface, with the hydrophilic–lipophilic balance (HLB) being the most widely used parameter. The HLB refers to the hydrophilic/lipophilic ratio of a surfactant molecule and is given by a dimensionless scale, where W/O emulsifiers exhibit an HLB value below 9, being predominantly lipophilic, and O/W emulsifiers exhibit an HLB value above 11, being predominantly hydrophilic [68]. It should be pointed out that not only surfactants can be used as interface stabilizers. Some systems are stabilized through the adsorption of fine solid particles at the oil–water interface, called Pickering emulsions [69]. Because the particles are closely packed, and due to the particle–particle interactions that occur, the stabilizing film between droplets can be quite rigid when compared to the stabilization using a surfactant. Therefore, this type of stabilization can provide enhanced stability of the droplets, providing a barrier to aggregation and coalescence [70].

#### 4.1.1. Oleogel-In-Water Emulsions

Lipid digestion has persisted as a forefront research focus, underlining the changes that the lipid’s physical state and interfacial structure induce on the digestion outcome. Regarding food applications, the suitability of oleogels to become a versatile tool depends not only on the gelation mechanism itself but also on the interaction with the surrounding matrix, which in most food products may be constituted by a water/aqueous phase. Guo et al. [71] focused on the variations that the addition of rice bran wax (RBX) to an O/W emulsion matrix incites on both the emulsion stability and the in vitro digestion. With an increase in the concentration of RBX, the melting point of the emulsions increased, and overall oil rigidity improved. The RBX crystals were formed within the oil droplets, which suggests that the length of the crystals was restricted by the interfacial film of the oil droplets. However, the presence of the crystals impacted emulsion stability, where higher concentrations of gelator led to partial coalescence of oil droplets. As for digestion, the presence of RBX effectively retarded lipolysis, although there was not a linear correlation with the gelator concentration. For high concentrations of RBX, the crystals pierced through the interface, exposing the oil and becoming ineffective in the retardation of lipid digestion. In short, the addition of an oleogelator to an O/W emulsion must be accomplished in an optimum concentration, in a way that the delayed lipolysis benefits are balanced with the stability needs [71].

Comparably, Munk et al. [72] targeted the behavior of EC in oil while being in contact with an aqueous phase. While approaching this situation, the authors followed a cold-temperature methodology and a hot-temperature methodology (above the melting point of EC). In both cases, the aqueous and oil phases were prepared independently, with the oil phase being prepared above the melting point in both cases. For the cold-temperature methodology, the oil phase was cooled down to room temperature and mixed with the aqueous phase, while for the alternate methodology, the mixing was performed above the melting point of EC. The hot-temperature methodology did not result in oleogel droplets, but rather in liquid oil droplets; an accumulation of EC in the surface of the oleogel was observed, forming a shell, while the interior of the shell was constituted by liquid oil. Adversely, the low-temperature methodology prompted the EC to act as an efficient oleogelator, opening up its potential use as a structurant in many O/W emulsion-based food products.

#### 4.1.2. Water-In-Oleogel Emulsions

The current studies on the structuring of the oil droplets in an O/W emulsion foresee a variation of the product’s characteristics, mainly at a microscopical level. In contrast, the structuring of the bulk oil phase in W/O emulsions, apart from microscopical changes, results in structural differences that are more evident to the consumer. This may be the reason why it is a significantly more scrutinized field rather than oleogel-in-water emulsions.

Lupi et al. [73] have prepared structured W/O emulsions by blending olive oil, Myverol (mainly composed of MAGs), and cocoa butter. By adjusting the oil/cocoa butter ratio, different rheological properties were obtained without changing the oil phase/aqueous phase ratio or the total emulsifier concentration. The structured oil phase entrapped the water droplets, similarly to the ‘pure’ oleogels, yielding a stable water-in-oil emulsion. Moreover, from a macroscopic point of view, the emulsion behaved like a solid under small deformations. The obtained product was compared to commercial margarine, and its potential as a solid fat substitute was proven [73]. Although this is a valid approach to successfully reduce the quantity of saturated fat in solid-like products through PUFA substitution, it is not ideal, since it still features a saturated fat crystalline network. Natural waxes have a lower index of saturated fat, which is why they can present a better alternative for the production of margarine-like spreads. Toro-Vazquez et al. [74] developed a water-in-oleogel emulsion based on candelilla wax and MAGs, with the premise in mind that, more than just an oleogelator, the candelilla wax may help to stabilize the water droplets. In fact, it was confirmed that it also behaved as an emulsifier, adsorbing at the oil–water interface and being beneficial to the stabilization of the water droplets.

Öğütçü et al. [75] suggested a mechanism of structuring olive oil W/O emulsions using beeswax, concerning previous work using waxes in ‘pure’ oleogels. Despite the water droplets’ sizes having increased after 90 days, the produced emulsions were stable, with the beeswax acting as a stabilizer along with the emulsifier Tween 80 as a droplet stabilizer. The overall conclusions of the study qualify them as an eligible alternative to margarine and spread-like products. RBX is also a natural wax, which is a value-added by-product of the rice bran oil refining process. Pandolsook et al. [76] hypothesized that it could be used as an efficient oleogelator for food applications, specifically as a margarine substitute in cookies. Both oleogels and W/O emulsions were prepared from RBX oleogels, and the cookies were produced by successfully substituting the margarine for the oleogelled emulsions at a rate of 50% and 100% of margarine content.

Fundamentally, for the oleogelation of a water-in-oil emulsion, the gelator must be combined with a suitable emulsifier, depending on the intended properties of the formulation. The water content of the formulation also influences the overall properties of the gelled emulsion, which is why Wijarnprecha et al. [77,78] tackled the topic of dispersed aqueous droplets acting as active or inactive fillers. These two situations were compared by using the same formulations of canola oil and RBX, altering only the type of emulsifier in each formulation. Another variable that was studied was the water content, to comprehend the effects on the water percentage in the formulations. When using monostearin, which is a MAG, the droplets behaved as active fillers, offering sites for interfacial nucleation and growth of RBX crystals, resulting in attractive interactions between the crystal network and water droplets. This induced an overall increase of the emulsions’ firmness, with water droplets playing an important role; water contents of as low as 50% were produced and remained stable for at least 2 months. At high water percentages, however, the shear-sensibility and crystal structure recovery were hampered [77]. Adversely, the use of polyglycerol polyricinoleate (PGPR) as an emulsifier prompted the water droplets to behave as inactive fillers, where water droplets decreased the firmness of the emulsions. There was no evidence of interactions between the PGPR and the crystal network; as such, the water did not improve the emulsion rigidity. On the contrary, the increasing water volume fraction decreased emulsion rigidity, indicating that the wax crystal network became weaker. This weakening was associated with the decrease in the RBX fraction rather than the water content itself. However, even with droplets acting as inactive fillers, the elastic modulus was similar to the corresponding control, where the water fraction is replaced by an equivalent fraction of the continuous oil phase. Considering this, it can be stated that kinetically stable oleogelled W/O emulsions can be generated, which is a leap forward in the feasibility of swapping oil/fat with a certain amount of water, while reaching similar rheological properties.

### 4.2. Double Emulsions

A double emulsion is an emulsion of a single emulsion. Due to their suitability as a tool for the delivery of bioactive compounds, double emulsions are actively used in the pharmaceutical industry as drug delivery vehicles. This type of emulsions allows for a slow release of the drug, and the time of release can be optimized according to the intended aim, by modulating the method of production [68]. These properties are not always attained by single emulsions, since these can be very susceptible to chemical degradation, which is where the multiple emulsification method can offer superior characteristics. There are two categories of double emulsions:

- oil-in-water-in-oil (O/W/O)—a continuous oil phase contains water droplets with smaller oil droplets dispersed inside them;
- water-in-oil-in-water (W/O/W)—a continuous water phase contains oil droplets with smaller water droplets dispersed inside them.

Currently, the gelation of oil in double emulsion systems focuses on W/O/W emulsions rather than O/W/O, which is why these are the only double emulsions discussed hereafter.

#### Water-In-Oleogel-In-Water Emulsions

This type of emulsion has great potential in food-related areas and has been tested in the delivery of probiotics and protection of aromas in foods [79,80]. The inclusion of aqueous droplets in the oil globules of fat products can be used to reduce the fat content in these products. The gelation of the oil phase in W/O/W emulsions can provide structure to the oil phase and help to prevent destabilization phenomena, such as the coalescence of water droplets within the oil phase. Additionally, double emulsions are prone to molecular transport of water or encapsulated compounds from one phase to another, which is very noticeable in low-viscosity oil phases [81]. Nelis et al. [81] monitored the water transport and permeation of manganese ions throughout the double emulsion, having observed that the presence of fat crystals in the oil phase effectively prevents the leakage of the internal water phase, therefore controlling and preventing the release of compounds from the internal water phase. This may be explained by the tortuosity of the path for molecular transport originated by the gel network, which reduces the permeability of the oil phase. The solid network also provided mechanical strength to the gel, opposing the osmotic forces.

One of the main constraints for the application of double emulsions for food purposes is the lack of food-grade emulsifiers, which are capable of stabilizing double emulsions. As such, Goibier et al. [82] proposed a mechanism that allows for double emulsion stabilization without using any surfactant. This feat is accomplished by the gelation of the oil phase through the addition of fat crystals, during the preparation of a single W/O emulsion. After crystallization of the bulk fat through a temperature decrease, the single emulsion was added to an external water phase. The solid fat ingredient, constituted mainly by triglycerides, stabilized the aqueous droplets and was able to impede coalescence. The process was rather versatile, and a plethora of different solid fat ingredients was applied, such as milk, cocoa, palm, and coconut.

The stabilization of double emulsions through fat crystallization has also been reported by Liu et al. [83]. They focused on the ability of the fat crystals to provide resistance to the osmotic stress and suggested its potential utility in a temperature-triggered release of active compounds from the internal phases. Essentially, it comprises a double emulsion prepared using hydrogenated soybean oil as the oil phase, which is a semi-solid oil. Above the melting point of the fat crystals, the system was prone to osmotic pressure effects, such as an increase in the internal water droplet size. As such, the creation of an emulsion that responds to a temperature change is feasible, and the integration of active compounds on the aqueous phase can be performed for a purposeful release, responding to a temperature stimulus.

**Figure 4:**
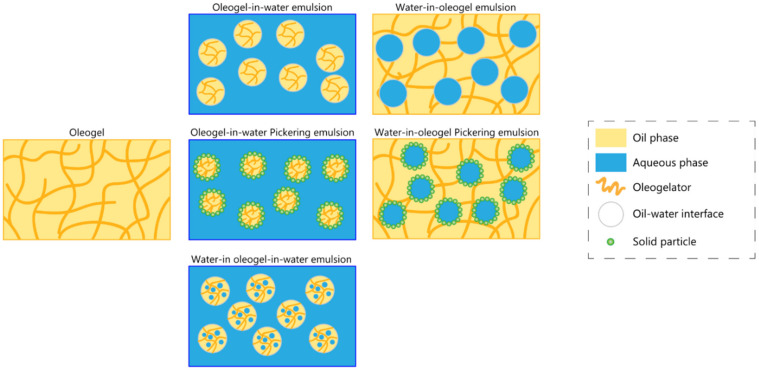
Schematic representation of different types of oleogel-based emulsion systems.

These oleogel-based emulsion systems feature very singular properties, and it is their versatility that makes them good candidates for different applications. Figure 4 displays a schematic representation of the structural organization of these types of formulation.

## 5. Oleogel-Based Systems as a Vehicle for the Delivery of Bioactive Compounds

As aforementioned, the structuring of liquid oils rich in PUFAs can bring significant benefits for human health, since they act as fat substitutes and have a much richer constitution than typical solid fats. Furthermore, the structure of oleogels constitutes a good matrix for the delivery of bioactive molecules, with it being both a way of protecting the integrity of bioactive compounds against oxidation or loss of functionality and a way of controlling their release. However, this area is still scarcely explored, and most of the studies rely on the integration of liposoluble compounds in the oleogel structure rather than hydrosoluble compounds. This seems to be the most straightforward approach due to the lipophilic nature of oleogels [57]. Oleogels also often serve as a starting point for the development of more complex structures, such as oleogel-based emulsions or oleogel-based Pickering emulsions, either to obtain additional benefits or to increase the formulation’s versatility. Table 1 presents some of the food-grade oleogel systems with proven effectiveness in the delivery of bioactive compounds.

**Figure 5:**
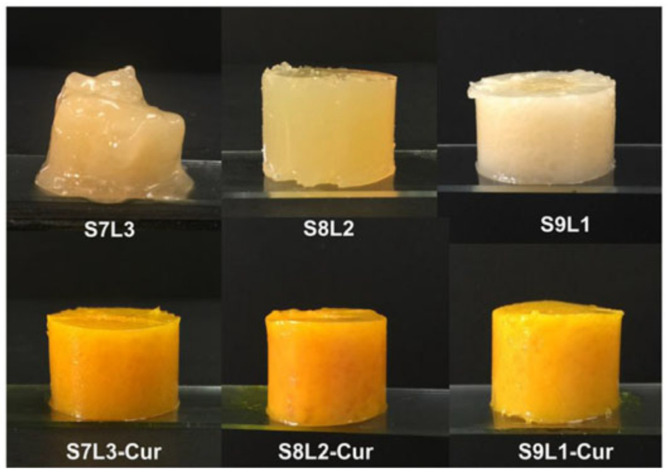
Oleogels developed with different ratios of β-sitosterol (S) and lecithin (L): 7:3, 8:2, and 9:1. The oleogels labelled with Cur are the ones loaded with curcumin. Reprinted from [86], with permission from Wiley.

Curcuminoids are a class of compounds that have been successfully studied in oleogels. Their water insolubility and rapid metabolism greatly affect their bioaccessibility and bioavailability, which hinders the reaping of its health-promoting benefits. Despite previous efforts to encapsulate curcuminoids, such as regular O/W emulsions, microemulsions, and solid lipid particles, the problem of its bioavailability was not explored thoroughly. The first oleogel system established for the delivery of curcuminoids dates from 2012 [84]. This study by Yu et al. encompassed a comparison between different oils and additives; in the end, the oleogel was prepared with medium-chain TAGs with added Span 20 and monostearin. This selection was based on the metastable solubility and bioaccessibility of the curcuminoids after lipolysis. Although the metastable solubility of the curcuminoids was not the highest on the medium-chain TAGs, after in vitro lipolysis, these were observed to generate higher bioaccessibility. The gelation process was proven not to affect the bioaccessibility, and oleogels were formed with a successful loading of 2.6% (*w/w*) of curcuminoids with a bioaccessibility of 80% in a fasted state. These oleogels were used for the fabrication of rapid-digestion emulsions, further proving that the delivery of poorly water-soluble nutraceuticals can be achieved through oleogel-based systems [85]. Since then, other types of formulations have been developed for the delivery of curcuminoids, with authors making use of the diversity of edible gelators suitable for oil structuring. Li et al. [86] developed a novel curcumin-loaded oleogel formulation, making use of the capability of β-sitosterol and lecithin to form self-assembled fibers, and studied its oxidative stability and release behavior. The developed structure protected the curcumin from being oxidized; on the other hand, a reciprocal effect was observed, where curcumin-loaded oleogels featured higher shelf-life stability when compared to non-loaded β-sitosterol + lecithin oleogels. The curcuminoids did not interfere with the gel network assembly, resulting in oleogels very similar in structure to the non-loaded oleogels. Figure 5 shows the oleogels developed with β-phytosterol and lecithin unloaded and loaded with curcumin.

**Figure 6:**
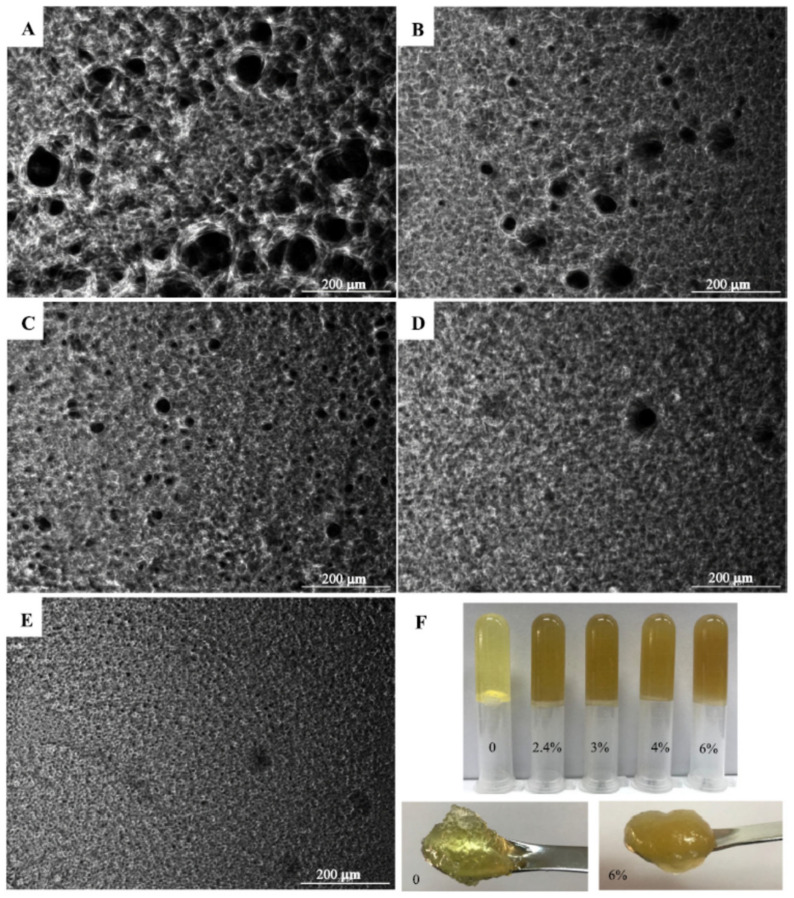
Microstructures (sorbitan monopalmitate content (**A**): 0, (**B**): 2.4%, (**C**): 3%, (**D**): 4%, (**E**): 6%) and macroscopic properties (**F**) of ethylcellulose (EC) oleogels. Sorbitan monopalmitate (SP) concentration corresponded to SP:EC ratios of 0, 1:5, 1:4, 1:3, 1:2. Reprinted from [89], with permission from Elsevier.

Vellido-Pérez et al. [87] designed a formulation comprising a fish oil concentrate as the lipid phase, aiming to stabilize and transport curcumin as a bioactive and the protection of the lipid phase. A powdered form of fully hydrogenated rapeseed oil with crystallization properties was used as a gelator in a concentration of 12% (*w/w*); both the gel structure and curcumin content in a minimum concentration helped to retard the oil oxidation. The oleogel structure and the manufacturing conditions were further optimized by a statistical experimental design and multi-response surface methodology. The curcumin content, the amount of gelator, and the manufacturing temperature were studied as independent variables, whereas the oxidation degree of the lipid matrix and the amount of loaded curcumin were selected using response surface methodology. The objective of this study was the simultaneous minimization of the lipid oxidation and maximization of loaded curcumin, with the optimum parameters for the studied variables being 0.150% (*w/w*) curcumin and 4.461% (*w/w*) oleogelator. Calligaris et al. [88] recently studied the effect of the oleogelator type and its resultant structure on oil lipolysis and the bioaccessibility of the curcuminoids during in vitro digestion. The liquid oil consisting of high oleic sunflower oil was enriched with turmeric extract, which is very dense in several curcuminoid types with well-known health benefits. Oleogels were prepared by mixing the enriched oil with 5% (*w/w*) of saturated MAGs, RBX, or a mixture of β-sitosterol and γ-oryzanol (2:3 *w/w*). Considering the rheology of the produced oleogels, all of them exhibited gel behavior, with the storage modulus being higher than the loss modulus. For a fixed frequency of 1 Hz, the RBX sample was shown to be the firmest, with the MAGs sample exhibiting the lowest storage modulus value. On the other hand, the β-sitosterol and γ-oryzanol samples demonstrated the highest yield stress value, which represents the value at which there is a structural breakdown of the gel. Regarding the bioaccessibility of the curcuminoids, the nature of the oleogelator significantly affected the outcome. The bioaccessibility of the curcuminoids included in the β-sitosterol and γ-oryzanol oleogel was comparable to the values referring to non-structured oil, suggesting that the gelator structure does not hinder the release of the bioactive molecules, and therefore does not compromise their bioaccessibility. On the other hand, the presence of crystalline networks (MAGs and RBX) presented lower bioaccessibility of the loaded curcuminoids, possibly due to interference of the crystalline particles with the bioactive compounds. The extent of lipolysis was also affected by the oleogelator options, and the lipid digestion was evaluated by measuring the free fatty acid release during the intestinal phase of the in vitro digestion, to which β-sitosterol and γ-oryzanol demonstrated the lowest extent. On the other hand, MAGs and RBX oleogels demonstrated lipolysis values closer to those reported for non-structured oil. In fact, different structural networks affect lipid digestion in different ways, possibly due to difficulties in lipase accessing the TAG digestion sites. The most up-to-date application of curcumin in an oleogel-based system was developed by Liu et al. [89]; the aim was to study the influence of a surface-active agent in the gelation process of ethylcellulose, in order to increase the loading of curcumin by reducing lipid oxidation and simultaneously improving curcumin solubility and chemical stability. At a morphological level, the samples prepared without the surface-active agent (sorbitan monopalmitate) had a wide distribution of pore size, and the gel network was very heterogeneous, with some areas being close to collapsing due to the connection of bigger pores. The addition of a small content of sorbitan monopalmitate reduced the number of large pores, an observation that was gradually noticeable with the increase in sorbitan monopalmitate content. This is evident in Figure 6, where the microstructures of the developed oleogels are shown. Evidently, with larger pores, there are bigger oil droplets entrapped within the network, which affects the macroscopic properties. The rheological data showed improved viscoelastic properties of the oleogels prepared with the surface-active agent, which might be associated with the interaction between the surface-active agent and the polar entities of the EC backbone. The results also showed that the creation of a more compact network had a direct influence on the inhibition of the formation of curcumin crystals, the slowing down of lipid oxidation, and resistance to UV radiation exposure.

Curcumin was also used by Ojeda-Serna et al. [90], in addition to two other poorly water-soluble compounds, betulin and quercetin, for a water-in-oleogel emulsion formulation, with the aim of increasing their bioaccessibility and cell permeability. The bioactive compounds were incorporated in the oleogel preparation, which was done using a MAGs blend as the gelator in a 10% (*w/w*) concentration. It was shown that not only the gelator can induce variations at the lipolysis and bioaccessibility level, but also the type of bioactive molecule influences these parameters, with distinct observations for each compound. Results showed that the use of emulsified oleogels enhanced the apparent permeability of betulin, which was not observed when curcumin and quercetin were used. On the other hand, regarding the bioaccessibility of each compound, the formulation was shown to enhance the bioaccessibility of quercetin but not of the other bioactive compounds. This was not the first oleogel formulation aiming at the delivery of quercetin, with Rocha-Amador et al. [91] focusing on the preparation of quercetin-loaded oleogels with canola oil, corn oil, and soybean oil. Quercetin is a flavonoid that can be widely found in most edible plants, typically in the glycoside-bound form. The authors aimed at assessing the effect of quercetin’s degree of glycosylation on its in vitro bioaccessibility. The results showed that, independently of the type of oil, lower bioaccessibility was observed for the higher degree of glycosylation. This might be associated with the impact of the glycoside group on the oleogel structure, which creates a more elastic and resistant network, hindering the release of the bioactive compounds. The results indicated an improved bioaccessibility of the quercetin when compared to bulk oil for both glycosylation states.

Another important bioflavonoid that was implemented in oleogel-based formulations is hesperidin, which has very low oral bioavailability due to its poor water solubility. Wei et al. [92] studied the integration of hesperidin in an oleogel-based Pickering emulsion stabilized by ovotransferrin fibrils, which were selected due to their high abundance and nutritional value. Hesperidin-loaded oleogels were prepared using soybean oil and monostearin, which were then used for the preparation of the Pickering emulsions. Both the lipolysis rate and bioaccessibility were proven to improve in the Pickering emulsion when compared to the oleogel. The ovotransferrin fibrils were also proven to be a very effective stabilizer at a very high internal phase volume ratio. Oleogel-based emulsions may also come up as a way of delivering nutraceuticals such as capsaicin, which is characterized by an intensely pungent flavor that results in a burning sensation; Lu et al. [93] developed a novel formulation that effectively alleviated the irritating effects of the capsaicin.

Carotenoids are a class of organic pigments that can be found in many fruits and vegetables, but also fungi, algae, and photosynthetic bacteria. Overall, carotenoids exhibit antioxidant properties, but individual carotenoids can display other characteristics. β-carotene (BC) is responsible for conferring a strong red-orange coloration to plant tissues and is also a precursor of Vitamin A. Vitamin A deficiency is a major health problem, especially in developing countries, and supplementation with Vitamin A is not an easy strategy to implement. As such, fortification of food with BC could be a viable alternative. Moreover, the consumption of a moderate dose of BC appears to have effects on eye health and cognitive performance, which may be associated with its antioxidant properties. Other than the dietary impact of BC itself, its ability to be converted to Vitamin A expands the ground of added health benefits, such as the improvement of immune function [108]. BC was first tested out as proof of principle for the capability of ethylcellulose oleogels to deliver bioactive compounds effectively by O’Sullivan et al. [94]. In this first approach, properties such as mechanical strength, in vitro digestibility, BC accessibility, and stability in the oleogel matrix were assessed, reinforcing oleogels’ value as carriers. These studies were performed using ethylcellulose’s physicochemical properties, particularly its viscosity, as a variable. The viscosities refer to the polymer molecular weight distribution, being commercially available in a range of viscosity values.

Other gelators such as MAGs have also been shown to be very effective for the delivery of BC. Fan et al. [95] developed both an oleogel and an oleogel-based emulsion for the delivery of BC. Although oleogel-based emulsions featured some benefits when compared to oleogels concerning the loaded amount, bioavailability, and biological activity of said compounds, little information was established for the case of BC. This work encompassed the preparation of oleogels using different liquid oils and the assessment of the BC accessibility in these oleogels; corn oil oleogels showed the most interesting results, which can be related to its length and unsaturation degree, serving as a starting point for the oleogel-based emulsion. The following step involved the assessment of different emulsifiers, specifically Tween 20, 40, 60, and 80, referring to an aliphatic chain of 12, 16, 18, and 18 carbons, respectively. Among the tested emulsifiers, Tween 20 facilitated the highest BC accessibility and extent of lipolysis, being chosen as the emulsifier for the nanoemulsions. Finally, the comparison between BC-loaded oil, oleogel, and oleogel-based nanoemulsions has revealed a positive tendency for both the bioaccessibility and extent of lipolysis, with the oleogel-based nanoemulsions showing the highest values. Moreover, the cellular uptake of BC-loaded in nanoemulsions was appreciably higher than in suspension, indicating that the emulsification process can improve the absorption of encapsulated BC. The results were also positive for the in vivo studies of bioavailability, which showed an increase in bioavailability of 11.5-fold compared to BC in bulk oil. Cui et al. [96] studied the effect of MAG content on the solubility and chemical stability of oleogels, by developing a corn oil-based oleogel, comprising different contents in MAGs. This allowed the obtaining of oleogels with different properties, and a positive relationship was observed between the concentration of MAGs and the strength of the gel network. Additionally, the solubility of BC was higher in the oleogels than in bulk oil, which is favorable for eventual food applications.

On the other hand, Martins et al. [97] developed a novel high oleic sunflower oil-beeswax system fortified with BC. The intent was to understand the structural implications of the addition of a compound such as BC in the formulation, encompassing polarized microscopy and rheological analyses, differential scanning calorimetry, wide-angle and small-angle X-ray analysis, oil binding capacity, oxidative stability, and color evaluation. Although the polarized microscopy observations did not exhibit relevant differences between non-loaded and loaded oleogels, the rheological analysis showed that the addition of the BC promoted changes in the crystallization process. The cooling process of the non-loaded oleogels happened more abruptly, with the cooling curve displaying a more pronounced ‘step’, possibly influenced by the heterogeneity of the beeswax composition. On the other hand, the loaded oleogels exhibited a more gradual increase of the viscoelastic properties during the cooling period and a less abrupt ‘step’, masking the effect of the heterogeneous beeswax structuring. The isothermal frequency curves showed the presence of a strengthened conformation in the oleogels prepared with BC, in comparison with that in oleogels prepared without BC. The X-ray results confirmed that the BC-loaded oleogels suffered dissimilarities at a structural level, affecting the positioning and size of the lamellar structures. The oil binding capacity is related to the capacity of beeswax crystalline structure to retain the oil, which helps to identify the relationship between oleogel strength and oil binding capacity. The observed tendency was that for low concentrations of beeswax, there is a clear improvement of the oil binding capacity with the addition of BC. While this type of relationship was not observed for higher concentrations of beeswax, the addition of BC did not hinder the oil binding capacity of the formulation. Qi et al. [98] also selected beeswax as an oleogelator in a novel oleogel-in-water Pickering emulsion approach for BC delivery. The study aimed at comparing the role of the beeswax versus a conventional Pickering emulsion (prepared without an oleogelator) on the oxidative stability and bioavailability of the BC. The oleogel-in-water Pickering emulsion demonstrated improved stability when subjected to a range of pH and salt concentrations and freezing-thawing stability, in addition to the enhanced chemical stability and bioavailability of the BC.

Although BC is the most widely studied carotenoid for food applications, other carotenoids such as lutein ester also possess strong health benefits. Jiang et al. [99] aimed at resolving one of the major problems with the stabilization of lutein ester in food applications, which is its poor light stability, by the oleogelation approach. By using monostearin as an oleogelator, it was concluded that the oleogel structure effectively prevented the degradation of lutein ester by UV irradiation, which was positively related to the content in monostearin.

One important property that should be modulated in the delivery of nutraceuticals via oral administration is the kinetics of the release, which is why oleogel structures are useful since they are capable of controlled release. Ferulic acid (FA) is known for its widespread use in anti-ageing creams, featuring strong antioxidant and anti-inflammatory properties. Its application in edible oleogels met its start in 2013 when an oleogel formulation was defined using olive oil as a vehicle. FA was submitted to a drastic acidic ambient to mimic stomach conditions, and the oleogel structure was capable of protecting the integrity of the FA to fulfil its nutraceutical function, with great rheological properties. In vitro release tests proved that the control samples, prepared without policosanol, were almost completely released after 2 h in stomach conditions, while by adding as little as 1% of policosanol, the delivery was controlled and delayed [100].

D-limonene is one of the main constituents of all citrus-derived essential oils, being GRAS for use as a flavoring agent and food preservative. However, it is very prone to oxidative degradation, causing the loss of its lemon-like flavor. As it is a hydrophobic compound, an oleogel-based approach seems very suitable for its protection in food products. Zahi et al. [101] developed a stable oleogel-based nanoemulsion using the method described by Yu et al. [85] with variations and performed an iterative method of selection of the oil phase, the emulsifier, and oleogelator. The final formulation, prepared with MCT oil, stearic acid, and Tween 80 at 10% (*w/w*), showed good stability in terms of Ostwald ripening and coalescence of the droplets during storage. Bei et al. [102] developed a novel system for the co-loading of D-limonene and nisin, both having reported antimicrobial activity. The preparation of an oleogel-based emulsion allowed for the dispersion of the D-limonene into aqueous phases in the form of small droplets, with an overall improvement of the antimicrobial properties. This approach may substitute current food preservative options as a more ’natural’ alternative.

Most of the above-described alternatives encompass an enrichment of the product’s nutritional level through the use of bioactive compounds. However, some authors focused on the aromatization of oleogels and oleogel-based emulsions, mostly envisioning their eventual applications as breakfast spreads and other similar products. Yilmaz et al. [103] focused on both the aromatization and the nutritional enrichment of the oleogels, developing an oleogel based on hazelnut oil and waxes. None of the included compounds undermined the gelation process, and their concentrations were intact after 3 months of storage. Through the performance of sensory descriptive analysis of the oleogels, parameters such as appearance, texture, aroma, flavor, and mouth-feel were assessed; this type of data is a first in the oleogel literature and is crucial for analyzing the acceptability of novel food products by the consumers. Chen et al. [104] developed a new strategy for the controlled release of volatiles with an oleogel-based emulsion approach, using a γ-oryzanol + β-sitosterol oleogelator system. The release behavior was registered under dynamic conditions using a self-designed model mouth cell, and the role of the microstructural characteristics on the volatiles’ release was assessed. The sitosterol adsorption to the droplet interface acted as an enhancer for the barrier properties of the droplet membrane, strengthening the barrier properties. Moreover, the co-operative self-assembly of the oryzanol–sitosterol molecules demonstrated its value in delaying the release of the volatiles to the interface. This constitutes an advantage over conventional emulsions, promising a system with an improved flavor profile in low-fat and sterol-rich food products. Yang et al. [105] also investigated the potential of the delayed release of volatiles in an oleogel formulation comprising a mixture of phytosterols and MAGs as the oleogelator.

Tea polyphenols are known for their antioxidant properties and free-radical scavenging capability. However, due to their low solubility in oils, their use in food products with high lipid content is hindered. Shi et al. [106] developed an approach that encompasses the inclusion of tea polyphenols in emulsion-based oleogels, to benefit from its properties for preservation of the oleogels during storage. To allow the dispersibility of the tea polyphenols in the oil matrix, initially, a stearic acid–surfactant–tea polyphenol complex was prepared through emulsification and lyophilization, which was later dissolved in liquid oil in different concentrations. The tea polyphenols’ antioxidant activity was comparable to chemically synthesized food additives, and this approach seemed to be effective in delaying the onset of oxidative rancidity, serving up as a good strategy for combining the potential of water-soluble ingredients with lipid-rich food products. This approach marked a very significant difference regarding previous works, where only hydrophobic compounds were studied for oleogel-based formulations. Andrade et al. [107] also developed a novel mechanism for the delivery of hydrophilic vitamins, in a double-emulsion approach. This system envisioned the co-delivery of both hydrophilic and lipophilic compounds with increased stability when compared to conventional double-emulsions, and the assessment of the double emulsion behavior during in vitro digestion. A solution containing water, ions, and Vitamin B_12_ was used as the internal phase; the oil phase was partially crystallized with the addition of trimyristin to soybean oil and using phytosterol and Vitamin D_3_ as bioactive compounds. The external water phase was prepared with water and ions for the maintenance of the osmotic and Laplace pressures between the two water phases. Comparing the double emulsions with and without a gelled oil phase, the first samples exhibited increased lipid digestibility and an extended release of the lipophilic bioactive compounds. Additional experiments were performed with a gelled internal aqueous phase, which was proven to affect the release behavior of the compounds. This study suggested that the double-emulsion approach is not effective in protecting the hydrophilic compounds dispersed in the water phase, unless the water phase is gelled, which garnered more positive results.

## 6. Conclusions

The last 5 years were prone to the development of oleogels for the delivery of bioactive molecules. Most of the works were focused on the use of oil-soluble compounds. Still, some examples exist of emulgels and bigels to deliver water-soluble compounds and co-delivery of oil and water-soluble compounds in the same system. In addition to the significant number of studies on their development, production, and characterization, some studies evaluate their degradation mechanism during digestion. This approach allows oleogel-based systems to be exploited for the release of bioactive compounds in the human gut. The future will be to produce tailor-made oleogel systems so that we can control the oleogel structure breakdown, the lipolysis rate control, and at the same time the bioaccessibility of bioactive compounds. New studies on co-delivery systems are lacking and should be performed so that their advantages as delivery systems for lipophilic and hydrophilic compounds are demonstrated. In addition, more in vivo studies are needed. The complete understanding of the absorption mechanisms of these bioactive compounds is of paramount importance and needs to be addressed in the following years.

## Figures and Tables

**Table 1:** Food-grade oleogel systems with proven effectiveness in the delivery of bioactive compounds.

Bioactive Compound(s)	Oil	Gelator	Gelator Conc. (%)	Type of Structure	Main Conclusions	Ref.
Curcumin	MCT Oil	MAGs	20	Oleogel	Increase in oral bioavailability of curcumin in both structures. The emulsions had faster lipolysis than the oleogels.	[84]
	MCT Oil	MAGs	20	Oleogel-based emulsion		[85]
	Corn Oil	β-sitosterol + lecithin	12	Oleogel	The curcumin did not interfere with the gel network assembling; its bioaccessibility at the intestinal level was enhanced in a fasted state.	[86]
	Fish Oil	Fully hydrogenated rapeseed oil	3–7	Oleogel	The gel structure and curcumin content helped to retard the oil oxidation.	[87]
	Sunflower Oil	Saturated MAGs, rice bran wax, γ-oryzanol + β-sitosterol	5	Oleogel	The nature of the oleogelator affected the bioaccessibility of the curcumin during in vitro digestion, which was higher in the β-sitosterol + γ-oryzanol oleogel. However, the extent of lipolysis was lower on this oleogel.	[88]
	Corn Oil	Ethylcellulose	12	Oleogel	The addition of a surface-active agent improved curcumin solubility and stability while reducing lipid oxidation.	[89]
Betulin, Curcumin, Quercetin	Canola Oil, Coconut Oil	MAGs	10	Oleogel-based emulsion	The bioaccessibility and permeability of the bioactive compound depend on the type of molecule and not only on the oleogel system.	[90]
Quercetin	Canola Oil, Corn Oil, Soybean Oil	MAGs	8	Oleogel	Oleogels prepared with canola oil featured better bioaccessibility of the loaded quercetin.	[91]
Hesperidin	Soybean Oil	MAGs	3	Oleogel–Pickering emulsion	Both lipolysis rate and bioaccessibility of hesperidin were improved in the Pickering emulsion regarding the oleogel.	[92]
Capsaicin	MCT Oil	Sucrose stearate S-370	20	Oleogel-based emulsion	Enhancement of the bioavailability of capsaicin and in vivo proof of the reduced irritability of the capsaicin.	[93]
β-carotene	Canola Oil	Ethylcellulose	10	Oleogel	Increased stability of β-carotene in the oleogel and protection against oxidation.	[94]
	Coconut Oil, Corn Oil, MCT Oil	MAGs	18.2	Oleogel, oleogel-based emulsion	Cellular uptake and bioavailability of β-carotene were higher in the emulsion than in control (liquid oil).	[95]
	Corn Oil	MAGs	10, 15, 20, 25	Oleogel	The oleogel structure improved the heat/light stability and solubility of β-carotene.	[96]
	High Oleic Sunflower Oil	Beeswax	2, 4, 6, 8	Oleogel	β-carotene improved the strength and oil-binding capacity of the oleogels; higher beeswax concentration improved oxidative stability of the oleogels.	[97]
	Soybean Oil	Beeswax	10	Oleogel–based Pickering emulsion	Improved pH/salt concentration/freeze–thaw stability; enhanced chemical stability and bioavailability of β-carotene.	[98]
Lutein Ester	Sunflower Oil	MAGs	4, 6, 8, 10, 12	Oleogel	The oleogel structure successfully protected lutein ester from UV radiation.	[99]
Ferulic Acid	Olive Oil	Policosanol	3	Oleogel	The addition of a gelator to the FA-loaded oil helped to control the release in stomach conditions.	[100]
D-limonene	MCT Oil	Stearic acid	5, 10, 15	Oleogel-based emulsion	Increased storage stability regarding conventional emulsions.	[101]
Nisin, D-limonene	Peanut Oil	Stearic acid	70	Oleogel-based emulsion	The combined use of D-limonene and nisin improved the antimicrobial properties and supported its use as a food preservative.	[102]
Volatile Aromas, Vitamins	Hazelnut Oil	Beeswax, sunflower wax	5	Oleogel	The addition of flavorings and vitamins did not undermine the gelation process and its concentration was intact after 3 months of storage.	[103]
Volatile Aromas	Sunflower Oil	γ-oryzanol + β-sitosterol	10	Oleogel-based emulsion	Successful delay of volatile release by entrapment in an oleogel network.	[104]
Volatile Aromas	Sunflower Oil	β-sitosterol + MAGs	10	Oleogel	The combination of 2 gelators resulted in stable oleogels, with controlled release of volatiles.	[105]
Tea Polyphenols	Peanut Oil	Stearic acid	5–30	Oleogel	The tea polyphenols helped to extend the storage stability of the oleogel.	[106]
Phytosterols, Vitamin D_3_, Vitamin B_12_	Soybean Oil	Trimyristin	15	Oleogel-based double emulsion	Higher extent of release of the bioactive compound and increased lipid digestibility, when compared to non-gelled double-emulsions.	[107]
